# Cooperative classrooms and prosocial behavior in primary education

**DOI:** 10.3389/fpsyg.2026.1725692

**Published:** 2026-02-11

**Authors:** Lucía-Fang León-García, Santiago Mendo-Lázaro, Víctor-María López-Ramos, Benito León-del-Barco

**Affiliations:** 1 Doctoral Program in Psychology, University of Extremadura, Cáceres, Spain; 2 Reseach Laboratory in Cooperation, Faculty of Teacher Training, University of Extremadura, Cáceres, Spain

**Keywords:** cooperative classrooms, cooperative learning, multilevel analysis, primary education, prosocial behavior

## Abstract

**Introduction:**

The practice of cooperative learning (CL) in the classroom has positive effects on performance and affective and social variables. Moreover, CL can be a methodology that favors the emergence of prosocial behaviors. In this research we intend to study the connection between CL as a contextual variable and prosocial behavior in Primary Education. We analyze the degree to which the five key components of CL (Social skills, group processing, promotive interaction, positive interdependence and individual accountability) explain and predict prosocial behavior among students.

**Methods:**

The sample was made up of 490 students between 10 and 12 years old complementing the Cooperative Learning Questionnaire (CLQ) to measure prosocial behavior using the Prosocial Behavior scale of the Strengths and Difficulties Questionnaire (SDQ).

**Results:**

The results show significant associations between prosocial behavior and Global Cooperation in the classroom (β = 0.0387; *p* < 0.001). Our results show that social skills (β = 0.194; *p* < 0.01), group processing (β = 0.095; *p* < 0.01) and individual responsibility (β = 0.091; *p* < 0.01) in a cooperative classroom are associated with students’ prosocial behavior.

**Discussion and conclusion:**

This study has demonstrated the relationship between cooperative classrooms and prosocial behavior. A cooperative classroom is an effective tool for giving and receiving social support. Interaction and group processing in cooperative classrooms enable students to develop social skills, which are a predictor of prosocial behavior. Cooperative classrooms make students more accountable, and this accountability has a positive impact on prosocial behavior. These findings represent a new contribution to the theoretical framework of CL and derive methodological implications for teaching practice. Therefore, it will be necessary to disseminate and motivate teachers to apply this methodology in the classroom.

## 1 Introduction

### 1.1 Cooperative learning

Cooperative learning can be defined from different theoretical perspectives. From a social psychology perspective, Deutsch (1949) defines a cooperative social situation as one in which the goals of separate individuals are so closely linked that there is a positive correlation between the achievement of their objectives. An individual only attains their goal if others also achieve theirs. From a behavioral perspective, Kelley and Thibaut (1969) define a cooperative structure as one in which the individual’s rewards or reinforcements are directly proportional to the quality of the group’s work.

Cooperative learning (CL), collaborative learning and other types of group learning are increasingly used in the classroom, all grade levels and school subjects with the aim of encouraging social acceptance among students, enhancing their performance and learning and allowing them to learn to work as part of a team and develop their social skills (Ginsburg-Block et al., 2006; León-del-Barco et al., 2017; Mendo-Lázaro et al., 2018). The ability to work cooperatively with others is vital for all students and represents a cornerstone of our society.

Cooperative learning, unlike other types of group learning, is characterized by a more structured methodology that comprises five key characteristics: positive interdependence, interaction, individual commitment, teaching of interpersonal and social skills, and quality of group processes (Johnson and Johnson, 2009). In cooperative learning the groups are based on a positive interdependence between group members. Objectives are structured in such a way that students take an interest in their own efforts and the performance of others. There is clear individual accountability, whereby each student’s mastery of the assigned content is evaluated. The group is given information about each member’s progress so that they know who needs help. Leadership and responsibility for learning are shared by all group members. Finally, the aim is to enable every group member to learn as much as possible.

Cooperative learning has been the subject of numerous research studies since the 1970s, when the first research into specific applications of this type of learning was published (Slavin, 1991). Some studies have focused on comparing three types of interaction and organization: cooperative, competitive and individual (Skon et al., 1981), concluding that cooperative situations are better in both academic and social terms. Most research has focused on analyzing the results and outcomes of CL techniques in relation to academic, social and affective variables. With regard to academic variables, the meta-analysis carried out by Johnson et al. (1981) showed that cooperation is superior to competition and individualism when it comes to the performance and productivity of all participants.

Cooperative learning also influences affective and social variables. In affective terms, CL techniques affect motivation and self-esteem. According to Slavin (1991), the most important psychological outcome of CL methods may be their effect on self-esteem. In social terms, the work in cooperative groups fosters acceptance among peers and makes a significant contribution to the development of social skills, the CL group functions as a social skills training group (Capodieci et al., 2019; Casal, 2016). Based on a meta-analysis, Johnson and Johnson (1990) conclude that, by far, cooperative learning situations produce more social support and lead to more positive attitudes toward those who are different. Social support not only facilitates more positive relationships between groups but is also linked to increased productivity. Other meta-analyses (Hattie, 2009; Johnson and Johnson, 2002; Roseth et al., 2008) have shown how CL has very strong effects on variables such as socialization, positive peer relationships and interpersonal attraction. Boix and Ortega (2020) conducted a review of the scientific literature to identify the benefits of CL in the core subject areas at primary level as an alternative to traditional, competitive educational methods. The benefits identified by the authors were primarily affective and social, with a positive influence on classroom relationships and environment. Van Ryzin et al. (2020b) in a study with students of color proves that CL improves positive relationships in peer relationships, reduces disparities and creates greater equity. Finally, CL is also an effective way to reduce school bullying and social exclusion (Polo-del-Río et al., 2017), as well as alcohol and tobacco consumption (Van Ryzin and Roseth, 2018).

### 1.2 Prosocial behavior

“Prosocial behavior” is a very broad term used to refer to a wide variety of different concepts (Pfattheicher et al., 2022). In general terms, it has been defined as behavior intended to benefit others by attempting to meet their emotional and physical support needs (Balabanian and Lemos, 2018). Auné et al. (2014) define it as a complex phenomenon that involves a person’s way of acting, which is shaped by their beliefs and feelings, and represents the way in which they orient themselves toward others by showing solidarity.

According to Martorell et al. (2011), prosocial behavior is a positive social behavior, which encompasses assistance, cooperation, exchange and adherence to social norms. One key component of prosocial behavior is its voluntary nature. It refers to positive social behaviors that are intended to benefit other people or groups (Martí-Vilar et al., 2019), improve personal and social adaptive behaviors (Carlo et al., 2014) and enable more harmonious coexistence with an individual’s close and immediate circle, such as family and friends (Sporzon and López-López, 2021).

For Carlo et al. (2014), prosocial behavior stabilizes during late childhood and early adolescence, declines in middle adolescence and increases again in late adolescence. Numerous studies have shown that adolescence is a critical period in social development, concluding that prosocial behavior is important when it comes to forming relationships with peers, as prosocial adolescents are predisposed to show empathy to others (Crone and Achterberg, 2022).

Studies on prosocial behavior or prosociality date back as far as the 1960s. Early research in this area associated prosocial behavior with an evaluation of the effort involved in helping others (Latané and Darley, 1970). Later studies have focused on analyzing the individual and contextual variables associated with prosocial behavior. With regard to individual variables, prosocial behavior has been linked to broader personality traits such as honesty, humbleness, kindness and empathy (Penner et al., 2005). According to Manesi et al. (2017), social value orientation and social mindfulness, which entail being open to the needs and desires of others in the present moment, are personality constructs that can be powerful triggers of prosociality. Other studies identify a positive association between problem-based coping strategies and prosocial behavior (Tur-Porcar et al., 2018).

With regard to contextual variables, the family is a social environment in which people learn to live alongside others from an early age and a key factor in the development of prosocial behaviors. Childhood is a stage in which the foundations for a person’s social development are laid and prosocial behaviors emerge or fail to do so (Arias Gallegos, 2015). A number of studies have highlighted the role of parenting styles in shaping children’s social development. A positive relationship with parents, based on reasoning, understanding, consensus and trust, led to prosocial behaviors among their children (Malonda et al., 2019).

Finally, schools are another environment in which prosocial behavior can be encouraged. Traditionally, socioemotional learning programs designed to promote social skills among students have been developed (Busching and Krahé, 2020; Caprara et al., 2015). However, several meta-analyses have shown that they have only a small impact on prosocial behavior (Taylor et al., 2017). These socioemotional learning programs are not carried out during the normal school day and a new approach must be found. There is no doubt that the use of CL methodologies in the classroom can encourage the emergence of prosocial behaviors. Research has confirmed that CL in the classroom improves prosocial behavior among students (Carrasco et al., 2018; Navarro-Patón et al., 2019).

According to Gillies (2016), cooperative learning affects students’ social and emotional development. We believe that key dimensions of CL, such as accountability, social skills, and group processing, are related to prosocial behavior. Accountability ensures that each student must work and strive for group achievement by helping their peers. On the other hand, social skills and group processing improve empathy, group reflection, active listening, and mutual respect among students and foster students’ willingness to collaborate and help their peers (Johnson and Johnson, 2014). These actions are closely linked to prosocial behaviors. Cooperative learning has a positive impact on students’ willingness to collaborate, help their peers, and be more aware of the needs of others, which fosters altruistic and collaborative behaviors (Yulianto et al., 2019). Other research has confirmed that cooperative learning in the classroom improves students’ social skills and prosocial behavior (Eisenberg and Fabes, 1991; Sharifi-Shayan et al., 2020; Solomon et al., 1990; Van Ryzin et al., 2020a).

### 1.3 Study objectives

Due to the limited number of studies linking CL to prosocial behavior and considerating the importance of prosocial behavior as a basic pillar of societal development and lie at the heart of all educational and social systems and how it is a protective factor against aggression, antisocial behavior and violent behavior (Barroso-Hurtado and Bembibre, 2019; Tur-Porcar et al., 2018), in this study aims to seek associations between the use of CL in the classroom and prosocial behavior among primary school children.

And, in more detail, we have set ourselves two objectives: 1. To verify the association between the dimensions of CL (Social Skills, Group Processing, Positive Interdependence, Promotive Interaction and Accountability) and prosocial behavior. 2. Analyze which dimensions of CL are associated with a high level of prosocial behaviors inside the classroom.

Our hypotheses were as follows:

1. There will be significant predictions between the dimensions of CL (Social Skills, Group Processing, Positive Interdependence, Promotive Interaction and Accountability) and prosocial behavior. 2. The dimensions of CL will be significantly associated with higher levels of prosocial behavior.

## 2 Materials and methods

### 2.1 Participants

The participants were selected using multi-stage cluster sampling and random selection of classes at schools with several groups in Years 5 and 6 at primary level. Cluster sampling was carried out by selecting 14 public educational institutions in Extremadura at random. For the random selection of class groups, all classes at the schools were assigned a number and random numbers were generated by a computer.

The sample was made up of 490 primary school students in Years 5 (222) and 6 (268). The average age was 10.56 years old (SD = 0.497, range 10–11); 56.3% (*n* = 276) were female and 43.7% (*n* = 214) were male. A total of 28 classes participated in the study.

### 2.2 Measures

#### 2.2.1 Cooperative learning questionnaire, CLQ

The CLQ (Fernández-Río et al., 2017) evaluates the key components of CL in the classroom. It is made up of 20 items divided into five dimensions: 1. Social skills, e.g., items such as “We work on discussing, debating and listening to others” and “We reach agreements within the group to make decisions.” 2. Group processing, e.g., items such as “We talk to each other to make sure that everyone in the group knows what is being done” and “Groupmates debate ideas and opinions.” 3. Positive interdependence, e.g., items such as “We cannot finish the tasks without the groupmates’ contributions” and “The better each group member completes his/her task, the better it is for the group.” 4. Promotive interaction, e.g., items such as “Groupmates relate with each other and interact during the tasks” and “Interaction among groupmates is necessary to complete the tasks.” 5. Individual accountability, e.g., items such as “Every group member has to participate in the group’s tasks” and “Every group member must strive to try hard in the group’s activities.”

These dimensions are evaluated through four items. The response format used is a five-point Likert-type scale (1 = Completely disagree to 5 = Completely agree). The CLQ provides a global cooperation factor, which is determined by the five factors.

The reliability indices for the different factors were: social skills factor, Cronbach’s α = 0.70, McDonald’s Ω = 0.71; group processing factor (α = 0.70, Ω = 0.71); positive interdependence factor (α = 0.65, Ω = 0.66); promotive interaction factor (α = 0.67, Ω = 0.68); individual accountability factor (α = 0.87, Ω = 0.88); global cooperation factor (α = 0.91, Ω = 0.91).

To prove whether the model found in the original validation study (Fernández-Río et al., 2017) is a suitable fit for our data, we used the goodness of fit indices shown in Table 1. As the table indicates, the fit indices are close to desirable values and show evidence of validity for the generalization of our findings.

**TABLE 1:** Goodness of fit indices for the proposed model, cooperative learning questionnaire (CAC) by Fernández-Río et al. (2017).

Model	χ^2^	χ^2^/*df*	GFI	IFI	TLI	CFI	RMSR	RMSEA
5 Factors	356.829	5.025	0.974	0.891	0.860	0.900	0.091	0.058

χ^2^, chi-squared test; χ^2^/df, chi-squared divided by degrees of freedom; GFI, goodness of fit index; IFI, incremental fit index; TLI, Tucker–Lewis index; CFI, comparative fit index; RMSR, root mean square residual; RMSEA, root mean square error of approximation.

#### 2.2.2 Strengths and difficulties questionnaire, self-report

The self-report version of the SDQ (Goodman, 1997) is a short instrument with excellent internal consistency in all its scales in both the international and the Spanish version. It comprises 25 items divided into five dimensions or subscales (1. Emotional Symptoms, 2. Conduct Problems, 3. Peer Relationship Problems, 4. Hyperactivity and 5. Prosocial Behavior). Each of the subscales is assessed via five items. The response format used is a three-point Likert-type scale (0 = No, not at all, 1 = Sometimes and 2 = Yes, always).

To evaluate the prosocial behavior variable in this study, we just used the Prosocial Behavior scale, which includes the items “I try to be nice to other people. I care about their feelings” and “I often volunteer to help others (parents, teachers, children).”

With regard to the overall reliability of the scale, it obtained Cronbach’s α = 0.76 and McDonald’s Ω = 0.76, while the Prosocial Behavior scale obtained α = 0.75 and Ω = 0.75. To determine whether the Prosocial Behavior scale is a suitable fit for our data, we used the goodness of fit indices shown in Table 2. As the table indicates, the fit indices are close to desirable values and show evidence of validity for the generalization of our findings.

**TABLE 2:** Goodness of fit indices for the prosocial behavior scale of the strengths and difficulties questionnaire (SDQ), self-report version.

Model	χ^2^	χ^2^/*df*	GFI	IFI	TLI	CFI	RMSR	RMSEA
1 Factor	20.125	4.025	0.985	0.914	0.824	0.912	0.079	0.040

χ^2^, chi-squared test; χ^2^/df, chi-squared divided by degrees of freedom; GFI, goodness of fit index; IFI, incremental fit index; TLI, Tucker–Lewis index; CFI, comparative fit index; RMSR, root mean square residual; RMSEA, root mean square error of approximation.

### 2.3 Procedure

Despite this being an investigation that causes no harm, given that it researches methods of classroom management in an educational context, we followed the ethical guidelines established by the American Psychological Association with regard to informed consent from parents, as all participants were underage. The study was conducted in accordance with the Declaration of Helsinki. Firstly, we contacted the schools to explain the study objectives and request authorization to complete the questionnaires. The questionnaire was then administered by class group. In this way, we were able to guarantee the anonymity of the responses, the confidentiality of the data obtained and the exclusive use of this data for research purposes. The questionnaires were administered in an appropriate setting without distractions, during school hours, taking around 20 min.

### 2.4 Design and statistical analysis

Taking into account the characteristics of the participants and the objective of the study, a cross-sectional *ex post facto* research design was followed, in which a phenomenon that has already occurred at a specific moment without continuity over time is studied and we do not manipulate the variables under study. Furthermore, an associative design was used, employing multilevel analysis, linear regression analysis and multinomial logistic regression, to study the predictive relationship between classroom cooperation and prosocial behavior. Finally, we used a questionnaire methodology for data collection.

First of all, a reliability analysis was performed on the instruments, and a confirmatory analysis was carried out.

(1) Regarding the first objective, a multilevel analysis and a linear regression analysis will be performed to verify the existence of significant predictions between the dimensions of CL (Social Skills, Group Processing, Positive Interdependence, Promotive Interaction and Accountability) and prosocial behavior.

The students participating in our study are grouped into different classes, whose characteristics may influence the dependent study variable: number of students per class, classroom management style, teacher, etc. The need to control this possible relationship between students and the classroom in which their learning activities take place prompted us to initially apply multivariate regression models for nested or hierarchical data. Hierarchical or multilevel linear models were designed to analyze data when some of the study variables are nested or grouped into higher level variables; in our study, students are nested in classes with their respective teachers. These models presuppose that students in the same class will tend to display similar behaviors.

After the multilevel analysis, the CL measured variables didn’t have a clear contextual character at the classroom level and to reaffirm the association between the CL elements, Social Skills, Group Processing and Accountability with prosocial behavior, we subjected the data to a more traditional statistical approach: Linear regression analysis and Multinomial regression analysis.

(a) Initially a multilevel analysis was conducted. The statistical adjustment process began with a random effects ANOVA model, which is known as the unconditional or null model. Once this first step was complete, two means as outcomes models (A and B) were fitted using regression analysis (RMR) in order to analyze how the explanatory study variables at the class level have a greater or lesser influence on prosocial behavior.

Firstly, Model A was fitted to ascertain the extent to which the global cooperation factor in the class explained and predicted prosocial behavior. Then, Model B was used to analyze the degree to which the five key components of CL (Social skills, group processing, promotive interaction, positive interdependence and individual accountability) explain and predict prosocial behavior among students.

In these analyses, the dependent variable was prosocial behavior. Global fit statistics (-2LL deviation, Akaike information criterion (AIC) and Bayesian information criterion (BIC) were calculated to determine the extent to which the proposed model is able to represent the variability observed in the data; the lower the value of the global fit statistics, the better the model fits the data.

(b) Multiple linear regression analysis was used to analyze which components of the Cooperative Learning significantly predicted the prosocial behavior of the participants. The dependent variable was Prosocial Behavior (PB). As independent variables, the five components of the CL: Social Skills (SS), Group Processing (GP), Positive Interdependence (PI), Promotive Interaction (PRI) and Accountability (A). The assumptions of normality, independence and collinearity were checked before performing the analysis.

(2) With regard to the second objective, a multinomial logistic regression will be performed to verify which dimensions of CL will be significantly associated with higher levels of prosocial behavior.

Lastly, to expand the information from the linear regression analysis, in order to understand how different predictors influence the probability of belonging to a high or low level of prosocial behavior and to estimate the odds ratio for each predictor, a multinomial logistic regression analysis was performed. In this analysis, the prosocial behavior (PB) was included as dependent variable, grouped into three levels, using a percentile criterion: low level (< 33%), medium level (between 33 and 66%) and high level (> 66%). The components of CL were included as independent and predictor variables. In summary, multinomial regression analysis gives us a deeper and more specific understanding of how independent variables influence the choice between multiple categories (high vs. low level of prosocial behavior).

Statistical analysis was carried out using the SPSS 26.0 package for PC and JASP Free.

## 3 Results

### 3.1 Variables analyzed in the research: descriptive statistics

Table 3 contains descriptive statistics for the study variables. The dependent variable was Prosocial Behavior and the predictor or explanatory variables for level 2 (class level) were Global Cooperation within the class, Social Skills, Group Processing, Positive Interdependence, Promotive Interaction and Accountability. The number of participants was *n* = 490 and the level 2 *n* = 28 classes.

**TABLE 3:** Descriptive statistics for the study variables.

Dependent variable (*n* = 490)	*M*	*SD*	Minimum	Maximum
Prosocial behavior (PB)	7.657	1.501	1	10
**Level 2 variables, class (*n* = 28)**	***M***	***SD***	**Minimum**	**Maximum**
Global cooperation factor (GCF)	3.874	0.562	1.65	5
Social skills (SS)	3.441	0.835	1	5
Group processing (GP)	3.585	0.805	1	5
Positive interdependence (PI)	3.991	0.687	1	5
Promotive interaction (PRI)	4.028	0.671	1	5
Accountability (A)	4.325	0.657	1	5

### 3.2 Hypothesis 1: Significant predictions between the dimensions of CL (social skills, group processing, positive interdependence, promotive interaction and accountability) and prosocial behavior: multilevel analysis and linear regression analysis.

Initially, an unconditional or null model was examined to estimate the variance between classes (level 2 variability) and within classes (level 1 variability). This model was calculated without level 2 (class) explanatory or contextual variables and serves as a reference point for evaluating the goodness of fit of the alternative models, in which explanatory or contextual variables at the class level are gradually incorporated. To do this, we applied random effects ANOVA (null model) to the data. Table 4 shows the results obtained, indicating that the estimate of the constant or intersection, the only fixed effects parameter in the model, was found to be different from zero. In other words, the estimated value (β = 13.149) of the Prosocial Behavior variable in the 28 classes that participated in the study was not zero (*p* < 0.001). Meanwhile, we observed the covariance parameter estimates, which are estimates of the parameters associated with the random effects of the model and found statistically significant differences (*p* < 0.001). The variance of the factor (classes β = 0.327) indicates the extent to which prosocial behavior varies between classes and the residual variance (residual β = 2.285) indicates the extent to which prosocial behavior varies within each class.

**TABLE 4:** Interrelations between prosocial behavior and cooperative elements in the classroom (Null model: one-factor random effects ANOVA; Models A and B: means as outcomes, regression analysis RMR).

Fixed effects	Null model	Model A	Model B
	β (SE)	β (SE)	β (SE)
Intercept: classes	13.149**(0.13)	10.366**(0.46)	9.689**(0.47)
Global cooperation factor (GCF)		0.0387** (0.01)	
Social skills (SS)			0.194**(0.03)
Group processing (GP)			0.095** (0.03)
Positive interdependence (PI)			0.046 (0.02)
Promotive interaction (PRI)			−0.010 (0.03)
Accountability (A)			0.091** (0.02)
**Random effects**	**β (SE)**	**β (SE)**	**β (SE)**
Residual	2.285**(0.15)	2.076**(0.14)	1.912**(0.13)
Classes	0.327**(0.13)	0.487**(0.18)	0.566**(0.19)
**ICC**	0.130	0.190	0.228
Explained variance (R^2)^		49%	73%
**Fit statistics**	**Value**	**Value**	**Value**
Deviation (-2LL)	1830.524	1800.913	1780.867
AIC criterion	1834.524	1804.913	1784.867
BIC criterion	1842.909	1813.294	1793.231

**p < 0.01. SE, standard error; ICC, intraclass correlation coefficient; Deviation, minus two times the logarithm of the maximum likelihood function; AIC, Akaike information criterion; BIC, Bayesian information criterion.

To interpret these values and calculate the variability between different classes in comparison with the variability between students in the same class, the intraclass correlation coefficient (ICC) was obtained. In our study, we found a value of 0.130; otherwise put, 13% of the total variability of prosocial behavior corresponds to differences between class means. These significant differences between class means constitute the level 2 variability.

Once the presence of differences between class means was confirmed, the next step was to identify any variables explaining these differences. To do so, two means as outcomes models were fitted using regression analysis (RMR).

Model A was fitted to ascertain the extent to which the Global Cooperation Factor variable explained prosocial behavior among the students. The results show significant differences in prosocial behavior according to the Global Cooperation Factor for the class (β = 0.0387; *p* < 0.001). The ICC rose from 13 to 19%. The proportion of explained variance at level 2 was.489 [(0.327–0.478)/0.327]. In other words, 49% of the differences in prosocial behavior observed between classes are attributed to the level 2 variable, Global Cooperation.

Model B was fitted, replacing the Global Cooperation Factor from the previous model with the five key components of CL to analyze the extent to which they each predict prosocial behavior among students. The data show significant differences in prosocial behavior for the variables Social *Skills* (β = 0.194; *p* < 0.01), *Group Processing* (β = 0.095; *p* < 0.01) and *Accountability* (β = 0.091; *p* < 0.01). The ICC was 22.8%. Meanwhile, the proportion of explained variance at level 2 was 0.730 [(0.327–0.566)/0.327]. In other words, 73% of the differences in prosocial behavior observed between classes can be attributed to the level 2 variables *Social Skills*, *Group Processing* and *Accountability*.

Model B is a better fit for the data, with lower values in the various information criteria (-2LL deviation, Akaike information criterion (AIC) and Bayesian information criterion (BIC). The higher ICC values in models A and B compared to the null model suggest the possibility of a traditional and standard data analysis. Perhaps the CL variables measured do not have a clear contextual character at the classroom level, for this reason, and to reaffirm the association between the CL elements, Social Skills, Group Processing and Accountability with prosocial behavior, we subjected the data to a more traditional analysis, a linear regression analysis.

In Table 5, we show the results of the linear regression analysis. The predictive model explains 9% of the variance in the prosocial behavior variable. The overall relationship between the model and the dependent variable is significant (*p* < 0.001). The most significant predictor variables were SS (β = 0.258, *p* < 0.001), A (β = 0.190, *p* < 0.001), and GP (β = 0.189, *p* = 0.009). Overall, the results obtained show that certain dimensions of cooperative learning in cooperative classrooms are significantly associated with prosocial behavior in participants. In particular, Social Skills (SS) and Accountability (A) emerged as the most consistent predictors of prosocial behavior. Also, Group Processing (GP) was significantly related to PB. In contrast, Positive Interdependence (PI) and Promotive Interaction (PRI) did not show significant associations in the regression model.

**TABLE 5:** Results of multiple linear regression models to predict prosocial behavior from the dimensions of cooperative learning.

(Predictor variables)	β (PB)	*p* (PB)	Tolerance	VIF
Social skills (SS)	0.258	<0.001	0.459	2.176
Group processing (GP)	0.189	0.009	0.359	2.787
Positive interdependence (PI)	0.137	0.064	0.407	2.456
Promotive interaction (PRI)	−0.098	0.143	0.421	2.377
Accountability (A)	0.190	<0.001	0.607	1.646

β, standardized coefficient Beta; VIF, Variance Inflation Factor.

### 3.3 Hypothesis 2: the dimensions of CL will be significantly associated with higher levels of prosocial behavior: multinomial logistic regression.

Finally, the multinomial regression analysis showed a satisfactory fit [χ^2^ = 40.008(10), *p* < 0.001; R Nagelkerke = 0.090] allowing a correct classification of 48.2% of the cases). As we can see in Table 6, the parameter estimates reveal that two dimensions are significantly and directly associated with the high level of Prosocial Behavior (PB): Social Skills (B = 0.193, *p* < 0.001) and Accountability (B = 0.118, *p* < 0.001). Likewise, the OR estimates of the model report that the probability of having a high level of Prosocial Behavior (PB) is 1.213 times higher in those participants who report Social Skills and 1.125 in those students who claim to be responsible.

**TABLE 6:** Results of multinomial logistic regression analysis for the prediction of Prosocial Behavior (PB) levels.

Predictor variables	High level prosocial behavior^1^				(Medium level prosocial behavior^1^)			
	B	OR	IC 95%		B	OR	CI 95%	
SS	0.193**	1.213	1.093	1.347	0.072	1.075	0.989	1.168
GP	0.078	0.925	0.832	1.029	0.065	0.937	0.854	1.029
PI	0.047	1.048	0.951	1.155	0.054	1.056	0.971	1.147
PRI	−0.069	0.933	0.851	1.023	−0.054	0.948	0.874	1.027
A	0.118**	1.125	1.045	1.210	0.044	1.045	0.989	1.104

SS, Social Skills; GP, Group Processing; PI, Positive Interdependence; PRI, Promotive Interaction; A, Accountability. Reference category:

^1^ Low level of Prosocial Behavior (PB).

**p < 0.001.

## 4 Discussion

This study aims to seek associations between the use of CL in the classroom and prosocial behavior among primary school children. And, in more detail, we have set ourselves two objectives: 1. To verify the association between the dimensions of CL (Social Skills, Group Processing, Positive Interdependence, Promotive Interaction and Accountability) and prosocial behavior. 2. Analyze which dimensions of CL are associated with a high level of prosocial behaviors inside the classroom.

In order to fulfill the study objectives and ensure that our results could be generalized, the reliability and validity of the Cooperative Learning Questionnaire (CLQ) and the Prosocial Behavior scale of the SDQ were first evaluated. The factor models found in the original studies were an adequate fit for our data. The indices were close to the optimal desired values, showing sufficient reliability and validity to allow our results to be generalized.

Our results confirm the associations between the use of CL in the classroom and prosocial behavior. It appears that helping and cooperative behaviors are promoted and developed in classes with more CL situations. These CL situations also enable moments of sharing and emotional support among students. These results are consistent with other studies that show that cooperative experiences increase the frequency of prosocial behaviors (Eisenberg and Fabes, 1991; Sharifi-Shayan et al., 2020; Solomon et al., 1990; Van Ryzin et al., 2020a).

Considering help and support for others, cooperation and empathy as three pillars of prosocial behavior, we reflected on why it is that CL situations encourage prosocial behavior. The CL techniques make a powerful contribution to developing and improving cooperation skills, especially among the participants most in need of group support. CL promotes greater interpersonal attraction between students and leads to more positive attitudes and emotions toward classmates. Students feel more loved, supported and accepted by others (Johnson and Johnson, 1990). Recent research has shown a positive correlation between levels of cooperation in the classroom and positive emotions such as trust, pride, enjoyment and tranquility (León-del-Barco et al., 2023). Cooperation is positively associated with empathy, which is the ability to situate oneself within the emotional perspective of others. In cooperative interactions, there is a change in student behavior as a result of them anticipating the needs and responses of other students. These changes enhance their ability to adopt other people’s cognitive and emotional perspectives. Numerous studies have found a positive correlation between empathy and prosocial behavior (Mestre Escrivá et al., 2002). This ability to situate oneself within the emotional perspective of others found in cooperative situations seems to be key elements in the development of prosocial (Kochanska, 2002).

Our results confirm our hypotheses. The data of multilevel analysis shows significant differences in prosocial behavior in terms of Global Cooperation and the Social Skills, Group Processing and Accountability variables. The model with the best fit to the data is Model B, in which the Global Cooperation Factor for the class was replaced by the five key components of CL. Model B showed that 73% of the differences in prosocial behavior observed between classes can be attributed to the level 2 variables Social Skills, Group Processing and Accountability, which is a very relevant finding. Prosocial behavior among students is less dependent on personal variables than on the learning methodology used, which was CL in this case. The more a cooperative approach is employed in the classroom, the greater the influence on prosocial behavior among students.

The data of linear regression analysis and multinomial logistic regression show that social skills, group processing and individual accountability in a cooperative class boost prosocial behavior among students. The probability of having a high level of Prosocial Behavior is 1.213 times higher in those participants who report that in the classroom they work on dialogue, agreements, listening, debate, ideas are represented and defended and 1.125 in those students who claim that in the classroom the members of the group participate, do their part of the work and make maximum effort in the tasks. These results corroborate previous findings that associate cooperative learning experiences with participation in a promotive interaction, validating the basic idea of social interdependence theory where the structure of the participants’ goals determines their interaction (Choi et al., 2011; Solomon et al., 1990). More positive peer relationships can support greater prosocial behavior (Van Ryzin et al., 2020a). However, our research represents a considerable advance by highlighting key elements in the interaction: group processing, social skills and individual accountability.

The group processing that takes place in learning situations brings group members into direct contact with others to discuss ideas, agree on decisions, encourage one another, offer help and assistance, etc. Group processing contributes to the development and improvement of social skills. Group members learn to request behavioral changes, ask for help, explain themselves, say no, voice criticism, defend rights, negotiate and challenge unfairness.

CL groups provide training in social skills (Ovejero, 2018). Working cooperatively, students imitate their peers (modeling), practice the interpersonal and communication skills they have learned (behavioral rehearsal, role play), receive immediate information about their behavior from their classmates (feedback) and generalize what they have learned in different situations.

Social skills in adolescence have been associated with participation in healthy pursuits, such as physical, musical and artistic activities (González Moreno and Molero Jurado, 2022). They have also been linked to emotional intelligence, self-esteem and self-concept (Tacca Huamán et al., 2020; Trigueros et al., 2020). Akelaitis and Lisinskiene (2018) conducted a study with a sample of adolescents and found that assertiveness is an important predictor of prosocial behaviors. In short, interaction and group processing in cooperative classrooms enable students to develop social skills, which are a predictor of prosocial behavior.

In CL situations, every group member commits to completing their share of the work and holds themselves accountable for achieving the objectives. Cooperative situations grant students greater responsibility and control over their learning, with greater autonomy and independence from the teacher (León-del-Barco et al., 2019). Accountability among group members is necessary for the group to be successful in completing the tasks assigned (Johnson and Johnson, 1990). Students must realize that they depend on one another and push themselves to do their very best. There is a clear individual responsibility, with all group members sharing responsibility for learning. Each group member undertakes to carry out their share of the work and the group is considered responsible for achieving the objectives.

We believe that participation in cooperative situations allows students to learn to be accountable and this accountability is key in prosocial behavior. Indeed, several studies have linked accountability to prosocial behavior (Hellmann et al., 2021; Gutiérrez-Sanmartín et al., 2011). In summary, a cooperative classroom makes students more accountable, and this accountability has a positive impact on prosocial behavior.

### 4.1 Limitations

There are several limitations to this study. The use of self-reports as a data collection method to assess cooperation in the classroom and prosocial behavior, as they are informed by students’ temporary, subjective perceptions. Other limitations include the cross-sectional study design, which makes it difficult to establish further inferences about the relationship between the study variables. Finally, the sample is limited to the Extremadura region of Spain. Ideally, the study should be replicated with a broader sample that is representative at the national level.

## 5 Conclusion

Prosocial behavior refers to actions that benefit others, including assistance, solidarity and cooperation. It is particularly important at times of crisis, when a society must tackle social, economic and political challenges that require the population to come together and cooperate. Organizations cannot function without cooperation between their different levels. The world is based on interdependence between people, communities and nations, making it necessary to help one another address the numerous political, economic, social and environmental challenges facing humankind.

Prosocial behavior, mutual assistance and cooperation are basic pillars of societal development and lie at the heart of all educational and social systems. Generally speaking, society praises and commends actions such as helping, cooperating and showing solidarity to benefit others or improve their quality of life. Meanwhile, people who display prosocial behavior feel more fulfilled and satisfied due to the selfless pleasure of serving others, as well as having better emotional and psychological adjustment and wellbeing. Moreover, prosocial behavior is a protective factor against antisocial and violent behavior.

It is vital to understand how prosocial behavior is manifested and how it can be developed and worked on in schools from an early age. This study has demonstrated the relationship between cooperative classrooms and prosocial behavior. A cooperative classroom is an effective tool for giving and receiving social support and building trust between peers to provide them with material and emotional support. Interaction and group processing in cooperative classrooms enable students to develop social skills, which are a predictor of prosocial behavior. Cooperative classrooms make students more accountable, and this accountability has a positive impact on prosocial behavior.

Therefore, it is important to inform and encourage teachers to apply CL methodology in the classroom. They must be made aware that a carefully designed program, interventions throughout the process and post-group work evaluation are necessary in order to access the multiple benefits of CL. Applying, knowing how to use and investing sufficient time in CL in the classroom will be highly fruitful and offer benefits and positive outcomes for students. The practice of CL in the classroom has a positive impact on performance, as well as on affective and social variables that directly influence prosocial behavior. The ability to work cooperatively with others is vital for all students and represents a cornerstone of our society. We must not forget that the leaders of the future, who will find solutions to the major challenges facing humankind and change the world through mutual assistance and cooperation, will be educated in our classrooms.

## Data Availability

The original contributions presented in this study are included in this article/supplementary material, further inquiries can be directed to the corresponding author.
